# Sex Differences in Age-Related Physical Changes among Community-Dwelling Adults

**DOI:** 10.3390/jcm10204800

**Published:** 2021-10-19

**Authors:** Takuhiro Okabe, Makoto Suzuki, Hiroshi Goto, Naoki Iso, Kilchoon Cho, Keisuke Hirata, Junichi Shimizu

**Affiliations:** Department of Rehabilitation, Faculty of Health Sciences, Tokyo Kasei University, 2-15-1 Inariyama, Sayama City 350-1398, Saitama, Japan; suzuki-mak@tokyo-kasei.ac.jp (M.S.); goto-h@tokyo-kasei.ac.jp (H.G.); iso-n@tokyo-kasei.ac.jp (N.I.); cho-k@tokyo-kasei.ac.jp (K.C.); hirata-ke@tokyo-kasei.ac.jp (K.H.); shimizu-j@tokyo-kasei.ac.jp (J.S.)

**Keywords:** aging, physical function, sex, middle-aged and older adults, rehabilitation

## Abstract

The prevalence of physical functioning limitations is positively correlated with age in both men and women. However, whether the appearance of deterioration differs depending on physical function and sex remains unclear. This study aimed to clarify the modes of age-related changes in physical function and sex differences in middle-aged and older adults. This study comprised 124 (46 men and 78 women) healthy adults aged 30 years or older and examined gender differences in physical function. The results of this study showed that one-leg standing time had the highest rate of age-related decline in both men and women, followed by knee extension strength, skeletal muscle mass, the 5 m walking test, and the timed up and go test. The sex-specific points showed a high rate of decline in trunk forward bending in men and a high rate of decline in forced expiratory volume (1 s) and gradual rate of decline in the bone area ratio in women. After middle age, it is desirable to start monitoring and training balance, muscle function, and walking. Men require early intervention for flexibility, and women require early intervention for respiratory function and continued intervention for bone mineral density.

## 1. Introduction

Age-related decline in physical functioning is a major factor in life disorders common in men and women. Physical functioning is an important marker of healthy aging and is a dynamic aspect of health. In Japan, the baby boomer population will reach its peak in 2025, and Japan will become a super-aging society in which one in four people is 75 years old or older. From the viewpoint of preventive medicine, under such circumstances, various fields, such as medical care, long-term care, and welfare aim to prevent age-related deterioration of physical functions, such as muscular strength, balance ability, and walking ability and extend healthy life expectancy.

Physical functions, such as muscle function [1,2], walking ability [2,3], flexibility [3], balance ability [4], respiratory function [5], and bone density [6], have been reported to decline with age.

Skeletal muscle mass begins to decrease from approximately 50 years of age and has been reported to decrease markedly more in the lower limbs than in the upper limbs [1,7]. Muscle strength peaks in middle age and by 90 years of age, declines by up to 50% [8]. Rapid walking speed decreases with advancing age, especially after 70 years [9], while balance ability is reported to decrease after the age of 40 [10]. The lung matures by 20–25 years of age (maximum lung function is reached at approximately 25 years in men and 20 years in women), after which aging is associated with a progressive decline in respiratory function [5]. In addition, peak bone mass is reached in early adulthood and decreases with age from approximately 50 years [11]. These age-related functional declines are factors in sarcopenia, frailty, and locomotive syndrome and cause inhibition of social participation, due to falls, fractures, and inactivity. However, the order in which each physical function declines is unclear.

Skeletal muscle decline due to aging has been reported as a sex difference in physical functioning, with age-related skeletal muscle mass loss in men twice as fast as in women [12]. In contrast, previous studies have reported that women have a longer life expectancy than men but often live longer with disabilities [13]. Compared to men, women have poorer levels of physical functioning [14,15] and experience a more rapid decline in functioning [16,17]. The prevalence of physical functioning limitations is positively correlated with age in both men and women. However, whether the appearance of deterioration differs depending on physical functioning and sex remains unclear. Therefore, it is not clear what preventive interventions should be administered to middle-aged and older adults. If we can identify the mode of change in physical functioning decline with age and sex, we may be able to contribute to effective interventions to prevent physical function decline. Therefore, regarding muscle strength, lower limb muscle strength may show a sharper decrease than upper limb muscle strength, and walking ability affected by these factors may show a gradual decrease. In addition, regarding sex differences, we hypothesized that men with more basic physical strength, but a shorter lifespan, might exhibit a more rapid decline. This study aimed to clarify the modes of changes in physical functioning and sex differences in middle-aged and older adults.

## 2. Materials and Methods

### 2.1. Research Design and Subjects

A cross-sectional study design was utilized in which samples were retrospectively extracted from the survey database for the Tokyo and Saitama regional areas from 2018 to 2020. This study comprised 124 healthy middle-aged and older adults aged 30 years or older (46 men, 78 women; mean age, 66.0 ± 12.0 years), who received an explanation of the purpose of this study and provided written consent to participate. The eligibility criteria were as follows: community-dwelling individuals; absence of palsy, knee pain, and injury; and no use of assistive devices for walking and sit-to-stand. The study was conducted according to the guidelines of the Declaration of Helsinki and was approved by the Research Ethics Committee of Tokyo Kasei University (SA2019-1, date of approval: 24 April 2019).

### 2.2. Physical Functioning Measurement

Height and weight were measured with the subjects wearing light clothing and no shoes. Body mass index (BMI) was calculated from height and weight as follows: weight/height squared (kg/m^2^). Physical functions, including skeletal muscle mass, vital capacity, bone area ratio, the 5 m walking test, timed up and go (TUG) test, trunk forward bending, grip strength, knee extension strength, one-leg standing time with eyes closed, and visual reaction time, were measured.

#### 2.2.1. Skeletal Muscle Mass

Skeletal muscle mass was measured using a bioelectrical impedance analyzer. Height was measured using a stadiometer (PA-200, UCHIDA YOKO Co., Ltd., Tokyo, Japan), and body weight and skeletal muscle mass were measured, using a body composition analyzer (InBody470; InBody Japan Inc., Tokyo, Japan). The bioelectrical impedance analysis (BIA) method is suitable for screening body composition, including muscle mass, because it is safe, simple, reliable, valid, and transportable, compared to computed tomography, magnetic resonance imaging, and dual-energy X-ray absorptiometry methods [18]. Each subject was barefooted, stood on the left and right metal plates, and grasped the metal conductor with both upper limbs for measurement. Quantitative evaluation of the skeletal muscle mass by the BIA method using Inbody is reliable and valid [19,20].

#### 2.2.2. Forced Expiratory Volume (1 Second)

Forced expiratory volume (1 s [FEV_1_]) was measured using a digital spirometer (AS-407, MINATO Medical Science Co., Ltd., Osaka, Japan). The subject held their nose and tried to exhale as forcefully and quickly as possible until all the air had been expelled. Subjects were instructed to continue exhaling during this stage. FEV_1_ is associated with physical activity [2,21,22], is a predictor of the risk of cardiovascular disorders and mortality [23], and is used to evaluate respiratory and circulatory functions.

#### 2.2.3. Bone Area Ratio

Bone density was examined along the heel bone, using quantitative ultrasound to measure the bone area ratio (Benus evo; Nihon Kohden, Tokyo, Japan). The ultrasound pulse reflection and transmission methods were used together. This method does not use X-rays, making it ideal for examining pregnant women and young people. Each subject sat in a chair, and measurements were taken on the right heel. Quantitative measurement of bone density using ultrasound is used as a valuable tool for osteoporosis screening [24,25].

#### 2.2.4. 5 m Walking Test

For the 5 m (meter) walking test, a distance of 3 m was set for the run-up, and the measurement started 3 m before the 5 m test distance. The measurement started when a part of the body crossed the 5 m start line and ended when the body crossed the 5 m goal line. The 5 m walking test was performed once at maximum walking speed and recorded in seconds. The 5 m walking test is a reliable evaluation tool used in large-scale surveys to evaluate walking ability [26].

#### 2.2.5. Timed Up and Go Test

For the TUG test, the subject stood up from an armless chair, walked 3 m, made a turn around a placed cone, walked back, and sat down again. The time from getting up from the chair to sitting down was measured. Subjects tried to walk as quickly as possible without shoes. The test was performed once and recorded in seconds. TUG is recommended as a regular screening test for falls in the American Geriatrics Society and the British Geriatrics Society guidelines [27], and is a reliable and valid assessment tool [28].

#### 2.2.6. Trunk Forward Bending

The purpose of the trunk forward bending measurement is to determine the degree of flexibility. The subject was placed in a long-sitting posture with the hips, back, and head close to a wall and arms outstretched front horizontally with the floor; the lumbar joint was bent forward, and measurement was recorded at the point reached by the fingertips. Measurements were taken once, without bending or recoiling the knees or extending one hand more than the other. The recorded unit was centimeters. Trunk forward bending is an index of flexibility and is a highly valid evaluation tool applied in physical fitness tests by the Ministry of Education, Culture, Sports, Science and Technology of Japan [29].

#### 2.2.7. Grip Strength

Grip strength of the dominant hand was measured, using a Smedley-type (mechanical) handgrip dynamometer (Smedley; Matsumiya Ika Seiki Seisakujo, Tokyo, Japan). To measure the grip strength, the dynamometer was held in a standing position with the pointer facing outward, and the width of the grip was adjusted so that the interphalangeal joint of the index finger was bent 90°. In an upright position with the feet hip-width apart, the arms were lowered naturally, and the dynamometer was grasped with maximum force without touching the body or clothing. Measurements were taken twice on the dominant side, and the average value was used for the analysis. The measurements were recorded in kilograms. Grip strength is a highly reliable and valid evaluation tool used in national surveys as a representative value of individual muscle strength [29,30,31].

#### 2.2.8. Knee Extension Strength

Knee extension strength was measured using a dynamometer (μTas-01, Anima Co., Ltd., Tokyo, Japan) for isometric maximal voluntary contraction of the predominant lower limb knee extensor. Subjects comfortably sat in a chair with their torso upright, maintaining a knee-to-hip angle of 90°. The task involved maximum knee extension while maintaining posture. Measurements were taken twice on the dominant side, and the average value was used for the analysis. The measurements were recorded in kilograms.

#### 2.2.9. One-Leg Standing

One-leg standing time was measured as an evaluation of balance. The time from the signal of “Please raise your foot” to one of the following conditions was measured: the position of the standing foot shifted, the raised foot touched the floor, or the raised foot touched the supporting leg. The upper threshold was set at 30 s. The measurement was performed once on each side with the eyes closed, and the average value was used for the analysis.

All tests were performed by an occupational or physical therapist and a trained research assistant.

### 2.3. Statistical Analyses

The *t* test was performed to compare each physical function between men and women. To verify the mode of change in physical function with aging, the score of each physical function was normalized, using the average score in the 30 s [29,32,33,34,35,36,37,38], and the linear model of Equation (1) used the generalized least squares method, where “t” denotes each person’s age, “α” denotes the physical function level of 30 s, and “β” denotes the rate of decline for each physical function:

f(t) = α + βt(1)

We approximated the measured normalized data, and the absolute values of β were compared. Statistical analyses were performed using the Statistical Package for the Social Sciences (S IBM SPSS Statistics for Windows, Version 26.0, Armonk, NY, U.S.A.) and R 3.5.2 software (R Foundation for Statistical Computing, Vienna, Austria).

## 3. Results

Table 1 shows the characteristics of the study subjects. Men had significantly higher skeletal muscle mass and stronger grip and knee extension strength than women. Women had significantly higher forward trunk bending measurements than men. There were no significant differences in age, BMI, FEV_1_, bone area ratio, the 5 m walking test, TUG test, or one-leg standing time between men and women.

**Figure 1:**
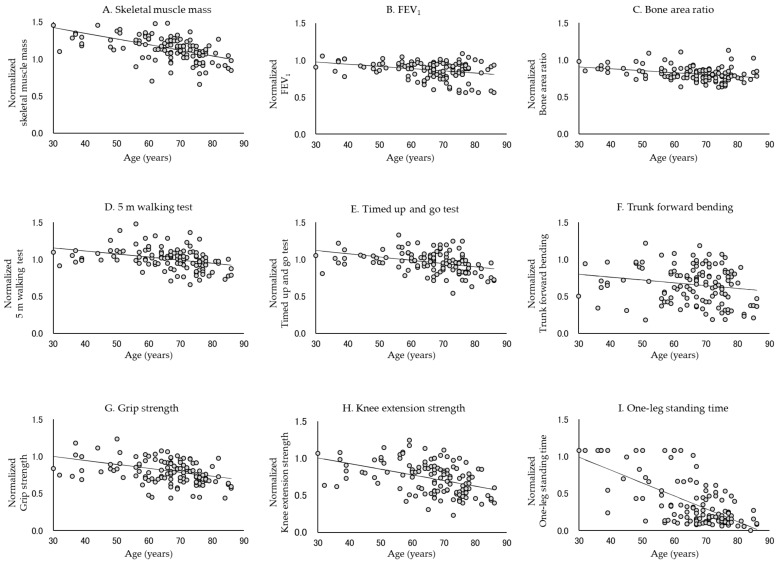
Normalized distribution and linear model of each physical function in all subjects. Each physical function declines with age. In particular, the rate of decrease in one-leg standing time, knee extension strength, and skeletal muscle mass was high.

**Figure 2:**
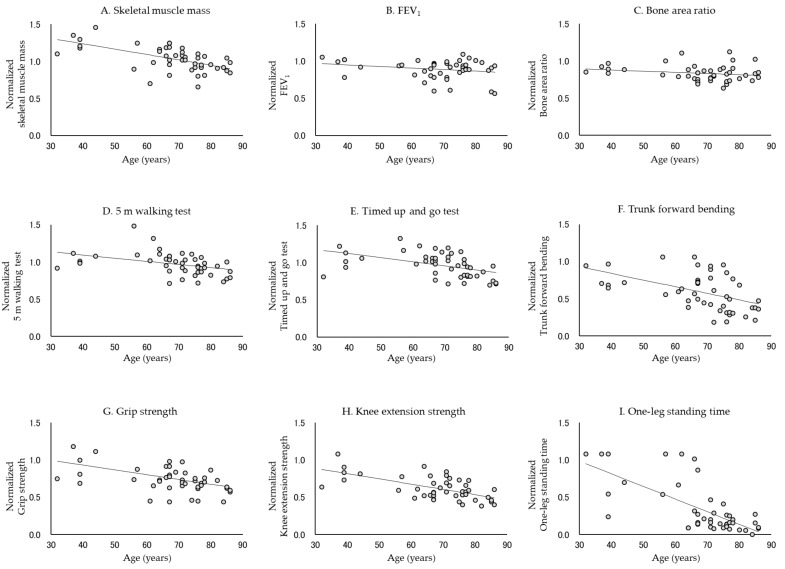
Normalized distribution and linear model of each physical function in men. Each physical function declines with age, except for FEV_1_ and bone area ratio. In particular, the rate of decrease in one-leg standing time, trunk forward bending, skeletal muscle mass, and knee extension strength was high.

**Figure 3:**
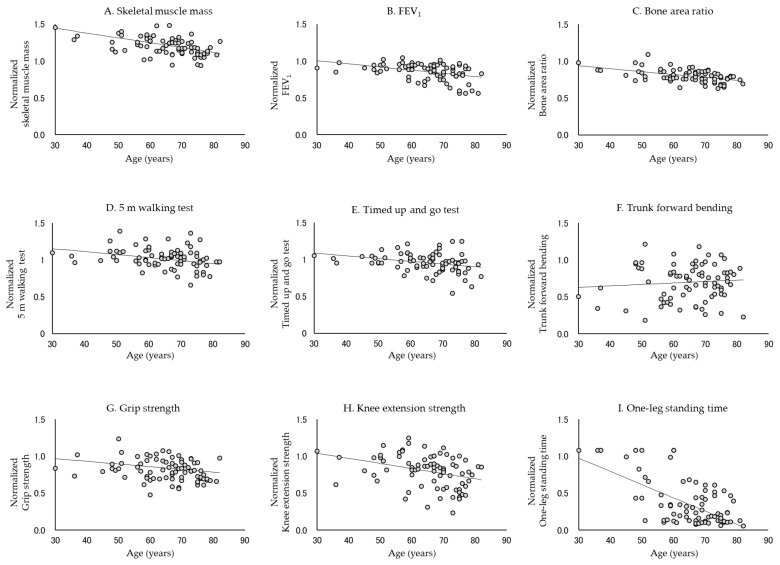
Normalized distribution and linear model of each physical function in women. Each physical function declines with age, except for trunk forward bending. In particular, the rate of decrease in one-leg standing time, knee extension strength, and skeletal muscle mass was high.

**Figure 4:**
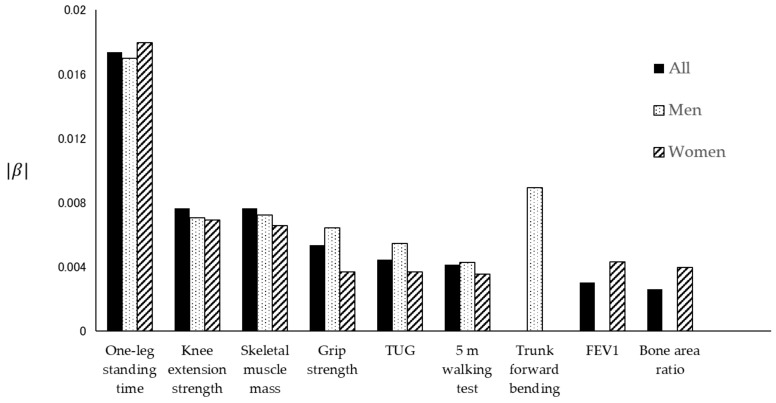
Comparison of the rate of decline in each physical function between all subjects, men, and women. Each bar shows the rate of decline in each physical function. The black bars represent all subjects, the dotted bars represent men, and the diagonally striped bars represent women.

Table 2 and Table 3 show the rate of decline in each physical function. In all subjects, the normalized data for each physical function approximated a linear model, except for trunk forward bending (*p* < 0.0001, Durbin–Watson ratio = 1.579–2.288). The absolute values of β were, in descending order, −0.0174, −0.0076, −0.0076, −0.0053, −0.0044, −0.0041, −0.0030, and −0.0026 for the one-leg standing time, knee extension strength, skeletal muscle mass, grip strength, TUG test, 5 m walking test, FEV_1_, and bone area ratio, respectively. In contrast, in men, the absolute values of β were, in descending order, −0.0170, −0.0089, −0.0072, −0.0071, −0.0064, −0.0054, and −0.0043 for the one-leg standing time, trunk forward bending, skeletal muscle mass, knee extension strength, grip strength, TUG test, and 5 m walking test, respectively. In women, the absolute values of β were −0.0180, −0.0069, −0.0066, −0.0043, −0.0039, −0.0037, −0.0037 and −0.0035 for the one-leg standing time, knee extension strength, skeletal muscle mass, FEV_1_, bone area ratio, TUG test, grip strength, and 5 m walking test, respectively. The normalized data did not approximate the linear model in FEV_1_ and bone area ratio for men or trunk forward bending for women. Figure 1, Figure 2 and Figure 3 show the standardized distribution of each physical function and its linear model for all subjects, men, and women. Figure 4 shows a comparison of the rate of decline in each physical function.

## 4. Discussion

This study showed that an age-related decrease in some physical functions was similar between men and women. In both men and women, the balance ability (one-leg standing time) had the highest rate of decline, followed by muscle function (knee extension strength, skeletal muscle mass) and walking ability (5 m walking test and TUG test). The sex-specific points were a high rate of decline in flexibility (trunk forward bending) in men and a high rate of decline in respiratory function (FEV_1_) and gradual rate of decline in bone mineral density (bone area ratio) in women.

The physiological and functional problems of muscles and the decrease in walking ability are related in a complex manner, and it seems that they have a significant effect on the decrease in balance ability. Muscle strength peaks in middle age and declines by up to 50% by 90 years [8]. The level of change with age may vary, due to several factors. For example, grip strength decline begins at age 40 [29], whereas rapid walking speed decreases significantly after 70 years [9]. These declines in physical function are expected to affect activities and participation levels, such as mobility, falls, and going out. The rate of decrease in one-leg standing time with eyes closed was the highest in both men and women. Balance ability is associated with muscle weakness [39] and flexibility [40] and is considered a comprehensive index of physical function. Age-related declines in physical function occur with diminished neuromuscular and musculoskeletal function, diminished muscle strength, and diminished coordination and motor control. Changes in sensory receptors and peripheral nerves associated with decreased visual acuity and vestibular function affect the lower extremities’ postural control and muscle output, resulting in decreased postural balance [39,41]. It has been reported that the one-leg standing test is useful for screening the risk of falls [42] and is an important evaluation for both young [43,44] and older adults [45]. It was suggested that one-leg standing time with eyes closed is useful as a factor that can detect early deterioration of physical function, even in middle-aged or healthy older adults.

Furthermore, both muscle strength and balance ability were reported to be significant predictors of walking disability [46], and balance ability, lower limb muscle strength, and walking ability were reported to be associated with fall risk and activities of daily living disorders [47,48]. Multimodal exercise, a complex program, has been reported to be more effective with multiple outcomes, including strength, balance, walking speed, and falls, compared to a single exercise. [49]. It is desirable to start training muscle strength and walking centering as balance training from middle age onward.

A comparison between men and women showed that trunk forward bending decreased in men, and FEV_1_ and bone area ratio decreased in women, which was sex specific. The flexibility of the trunk is lower in men than in women, and the decrease in flexibility seems to be faster in men than in women. It was reported that women are more flexible than men in both young and old age [50,51], and this study showed similar results. In addition, the fact that the decrease in bone mineral density was more pronounced in women than in men was consistent with the results of previous studies [6]. Previous studies have reported age-related declines in respiratory function in both men and women [52], and lung capacity values of women were significantly lower than those of men [53]. The lungs reach maturity by the age of 20 and achieve maximum function at about 25 years in men and 20 years in women. Lung function changes minimally and stabilizes between the ages of 20 and 35, after which it begins to decline [5]. However, Sharma et al. reported that the effects of aging on lung function vary significantly. In addition, age-related decline in FEV_1_ may have a non-linear phase with an accelerated rate of decline after the age of 70 [5]. In the future, we would like to examine the appropriate intervention timing and method to prevent these declines in physical function.

Notably, although there was no difference between men and women in the univariate analysis (FEV_1_, bone area ratio), there was a difference in the rate of decrease (β). Therefore, when comparing sex differences, it was suggested that not only cross-sectional numerical comparisons, but also changes due to aging should be investigated. Based on our results, balance ability and muscle function in both men and women, flexibility in men, and respiratory function in women should be evaluated and addressed from an early stage.

This study has several limitations. First, the individual physical functions were measured cross-sectionally. Therefore, changes in these individual parameters over time could not be considered. In addition, the subjects of this study were healthy, middle-aged, older adults. Therefore, the results of this study cannot be generalized to frail, older adults.

The result of this study clarified that physical function showed a sex-specific decrease. This result contributes to appropriate timing and sex-based interventions for middle-aged and older people.

## 5. Conclusions

Balance ability had the highest rate of age-related decline followed by muscle function and walking ability in both men and women. The sex-specific points showed a high rate of decline in flexibility in men and a high rate of decline in respiratory function and gradual rate of decline in bone mineral density in women. After middle age, it is desirable to start monitoring and training balance, muscle function, and walking. In addition, men require early intervention for flexibility, and women require early intervention for respiratory function and continued intervention for bone mineral density. The findings from this study provide useful information for the development of effective early interventions that aim to extend the healthy life expectancy of men and women in an aging society.

## Figures and Tables

**Table 1:** Characteristics of the study subjects.

	All *n* = 124	Men *n* = 46 (37.1%)	Women *n* = 78 (62.9%)	*p* ^†^
	Mean ± SD			
Age (years)	66.0 ± 12.0	68.0 ± 13.7	64.9 ±10.7	0.159
Body mass index (kg/m^2^)	22.8 ± 2.8	23.1 ± 2.1	22.6 ± 3.1	0.284
Skeletal muscle mass (kg)	22.1 ± 4.6	26.5 ± 4.3	19.6 ± 2.4	*
FEV_1_ (%)	74.4 ± 10.0	74.3 ± 10.5	74.4 ± 9.9	0.993
Bone area ratio (%)	27.0 ± 3.1	27.6 ± 3.5	26.7 ± 2.8	0.117
5 m walking test (m/sec)	1.93 ± 0.27	1.97 ± 0.30	1.91 ± 0.25	0.188
Timed up and go test (sec)	5.9 ± 0.9	5.9 ± 1.0	5.9 ± 0.9	0.976
Trunk forward bending (cm)	28.2 ± 10.7	25.0 ± 10.5	30.0 ± 10.5	0.012
Grip strength (kg)	28.1 ± 8.0	35.1 ± 8.0	26.4 ± 7.3	*
Knee extension strength (kg)	29.7 ± 8.8	34.7 ± 8.7	26.7 ± 7.3	*
One-leg standing time (sec)	10.1 ± 8.9	9.5 ± 9.4	10.4 ± 8.6	0.558
				*t*-test

SD: standard deviation, ns: not significant. ^†^ Significant difference between men and women in each physical function, * *p* < 0.001.

**Table 2:** Comparison of the rate of decline in each physical function in all subjects.

	All						
	β	α	DW	*p*	R^2^	*p*	R
One-leg standing time	−0.0174	1.51	2.031	*	0.427	*	1
Knee extension strength	−0.0076	1.24	1.769	*	0.149	*	2
Skeletal muscle mass	−0.0076	1.65	1.579	*	0.208	*	3
Grip strength	−0.0053	1.16	2.119	*	0.171	*	4
Timed up and go test	−0.0044	1.25	2.288	*	0.201	*	5
5 m walking test	−0.0041	1.28	1.97	*	0.114	*	6
FEV_1_	−0.0030	1.07	1.93	*	0.085	*	7
Bone area ratio	−0.0026	0.98	1.877	*	0.096	*	8
Trunk forward bending	−0.0038	0.91	1.792	0.060	0.025	0.043	

*β*: rate of decline, *α*: physical function level of the 30 s, DW: Durbin–Watson ratio, R: rank based on *β*, * *p* < 0.001.

**Table 3:** Comparison of the rate of decline in each physical function between men and women.

	Men							Women						
	β	α	DW	*p*	R^2^	*p*	R	β	α	DW	*p*	R^2^	*p*	R
One-leg standing time	−0.0170	1.49	2.141	*	0.493	*	1	−0.0180	1.54	1.971	*	0.375	*	1
Knee extension strength	−0.0071	1.10	2.303	*	0.466	*	4	−0.0069	1.25	2.046	0.002	0.105	0.002	2
Skeletal muscle mass	−0.0072	1.52	2.17	*	0.381	*	3	−0.0066	1.64	2.111	*	0.227	*	3
Grip strength	−0.0064	1.19	2.502	*	0.382	*	5	−0.0037	1.08	2.143	0.011	0.071	0.010	7
Timed up and go test	−0.0054	1.34	2.033	*	0.249	*	6	−0.0037	1.20	2.545	*	0.165	*	6
5 m walking test	−0.0043	1.26	1.814	0.010	0.120	0.011	7	−0.0035	1.25	2.135	0.008	0.072	0.010	8
FEV_1_	−0.0021	1.03	2.079	0.105	0.019	0.179		−0.0043	1.13	1.952	*	0.152	*	4
Trunk forward bending	−0.0089	1.20	2.419	*	0.288	*	2	0.0020	0.57	1.821	0.484	0.001	0.613	
Bone area ratio	−0.0016	0.94	1.907	0.192	0.021	0.168		−0.0039	1.06	2.214	*	0.253	*	5

*β*: rate of decline, *α*: physical function level of the 30 s, DW: Durbin–Watson ratio, R: rank based on *β*, * *p* < 0.001.

## Data Availability

Not applicable.
